# GLP-2 Prevents Neuronal and Glial Changes in the Distal Colon of Mice Chronically Treated with Cisplatin

**DOI:** 10.3390/ijms21228875

**Published:** 2020-11-23

**Authors:** Patrizia Nardini, Alessandro Pini, Anne Bessard, Emilie Duchalais, Elena Niccolai, Michel Neunlist, Maria Giuliana Vannucchi

**Affiliations:** 1 Histology and Embryology Research Unit, Department of Experimental and Clinical Medicine, University of Florence, 50139 Florence, Italy; patrizia.nardini@unifi.it (P.N.); alessandro.pini@unifi.it (A.P.); elena.niccolai@unifi.it (E.N.); 2 Inserm, TENS, The Enteric Nervous System in Gut and Brain Diseases, IMAD, University of Nantes, 44035 Nantes, France; anne.bessard@inserm.fr (A.B.); emilie.duchalais@gmail.com (E.D.); michel.neunlist@univ-nantes.fr (M.N.)

**Keywords:** antineoplastic drug, pleiotropic intestinal hormone, myenteric plexus, colonic mucosal layer, neuronal nitric oxide synthase (nNOS), choline acetyltransferase (ChAT), vasoactive intestinal peptide (VIP)

## Abstract

Cisplatin is a chemotherapeutic agent widely used for the treatment of solid cancers. Its administration is commonly associated with acute and chronic gastrointestinal dysfunctions, likely related to mucosal and enteric nervous system (ENS) injuries, respectively. Glucagon-like peptide-2 (GLP-2) is a pleiotropic hormone exerting trophic/reparative activities on the intestine, via antiapoptotic and pro-proliferating pathways, to guarantee mucosal integrity, energy absorption and motility. Further, it possesses anti-inflammatory properties. Presently, cisplatin acute and chronic damages and GLP-2 protective effects were investigated in the mouse distal colon using histological, immunohistochemical and biochemical techniques. The mice received cisplatin and the degradation-resistant GLP-2 analog ([Gly2]GLP-2) for 4 weeks. Cisplatin-treated mice showed mucosal damage, inflammation, IL-1β and IL-10 increase; decreased number of total neurons, ChAT- and nNOS-immunoreactive (IR) neurons; loss of SOX-10-IR cells and reduced expression of GFAP- and S100β-glial markers in the myenteric plexus. [Gly2]GLP-2 co-treatment partially prevented mucosal damage and counteracted the increase in cytokines and the loss of nNOS-IR and SOX-10-IR cells but not that of ChAT-IR neurons. Our data demonstrate that cisplatin causes mucosal injuries, neuropathy and gliopathy and that [Gly2]GLP-2 prevents these injuries, partially reducing mucosal inflammation and inducing ENS remodeling. Hence, this analog could represent an effective strategy to overcome colonic injures induced by cisplatin.

## 1. Introduction 

Gastrointestinal (GI) toxicity is a common complication of platinum-based drug chemotherapy. Nausea, vomiting, constipation and diarrhea are likely to occur within a few hours/days following the treatment (acute toxicity) and can persist up to 10 years after the treatment has ceased (late/chronic toxicity) [1]. The GI toxicity leads to malnutrition, dehydration and consequently rapid weight loss, influencing the patient’s quality of life [2,3]. In the worst cases, it can lead to delay in treatment cycles and dose reduction that, therefore, can impact the patient’s response to treatment and risk of relapse. 

Among platinum compounds, cisplatin, one of the most effective and widely used drugs in standard treatments of several solid cancers, causes early and late GI side effects. Cisplatin-induced mucosal damage likely has a significant role in the acute toxicity, whereas the pathophysiology of the cisplatin-induced chronic long-lasting side effects is still unclear [2,4]. 

Emerging evidence suggests that the persistence of cisplatin-induced GI side effects might be the result of a combination of intersecting mechanisms, including inflammation, secretory and motor dysfunctions resulting from pro-apoptotic actions of the drug upon the epithelium but also upon the enteric nervous system (ENS) [5,6,7]. Indeed, studies reported that chronic treatments with cisplatin or oxaliplatin cause a significant remodeling of the ENS, affecting the myenteric plexus in different GI segments [8,9,10,11,12,13,14,15]. In particular, platinum-based treatments were shown to increase the proportion of nNOS-immunoreactive (IR) neurons and S100β-IR enteric glial cells (EGCs) within the rat and mouse ileum and distal colon but decreased the proportion of ChAT-IR cells as well as the expression of GFAP-IR. This remodeling was correlated with impairment of rat ileum [13] and mouse distal colon motility [9,10]. Thus, preventing or counteracting the ENS changes induced by platinum-based treatments might be of interest to prevent their long-lasting side effects.

Glucagon-like peptide 2 (GLP-2) is a hormone synthesized and secreted by the enteroendocrine “L” cells of the ileum and colon in response to nutritional, hormonal and neural stimulation [16]. The actions of GLP-2 are transduced by the GLP-2 receptor (GLP-2R), a G-protein coupled receptor mainly expressed in the GI tract and central nervous system [16]. Even though the cellular distribution of this receptor remains controversial, the presence of the GLP-2R has been found in the enteric neurons, myofibroblasts and subsets of enteroendocrine cells [17,18,19,20,21]. In physiological conditions, GLP-2 exerts multiple beneficial effects on the intestine, including the maintenance of mucosal integrity, energy absorption and modulation of motility [22,23,24,25]. In several animal models of intestinal injury such as ischemia-reperfusion injury and chemically induced enteritis/colitis, a GLP-2 analog has been shown to exert trophic/reparative effects via antiapoptotic and pro-proliferating pathways [26,27,28]. Further, the administration of the native GLP-2 in animal models of inflammatory bowel disease had significant anti-inflammatory effects, reducing colonic inflammation via release of VIP [29,30]. Finally, a degradation-resistant GLP-2 analog (teduglutide) has been recently approved by European Medicines Agency and the US Food and Drug Administration as an innovative adjuvant treatment for short bowel syndrome [31]. For all these properties, mainly exerted on intestinal mucosa, there has been intense interest in the ability of GLP-2 to prevent chemotherapy-induced mucositis [26,27,32] but little is known about its protective effects upon chemically induced ENS remodeling.

GLP-2 exerts neuroprotective effects upon myenteric neurons in vitro, enhancing both neuronal survival in primary cultures and counteracting neuronal cell death induced by mast cell co-cultures [33]. Moreover, GLP-2 enteric neuroprotection was confirmed in vivo by Pini et al. [12], highlighting that the co-administration of GLP-2 analog during a long-term cisplatin treatment prevented gastric fundus myenteric neuropathy. However, whether GLP-2 protective abilities observed in the fundus also occur in the colon and whether the GLP-2 neuroprotective properties also affect EGCs currently remain unknown. 

Hence the present study investigated whether the co-administration of the GLP-2 stable analog protects the distal colon from the injuries induced by cisplatin chronic administration. Combining histological, immune-histochemical and biochemical approaches, we characterized the cisplatin damage (i) upon mucosal integrity and inflammatory response, likely responsible for the acute symptoms frequently observed during the treatment, and (ii) upon the ENS phenotypic changes accountable for the long-term symptomatology following chemotherapy interruption. In parallel, we evaluated the ability of the degradation-resistant GLP-2 analog, used in combination with cisplatin, to protect the colonic wall, preventing or reducing the mucosal damages and the ENS remodeling.

## 2. Results

### 2.1. GLP-2 Does Not Affect the Weight Loss Induced by Chronic Cisplatin Treatment

We first aimed at characterizing the impact of cisplatin and co-administration of GLP-2 upon the evolution of weight loss. 

**Figure 1:**
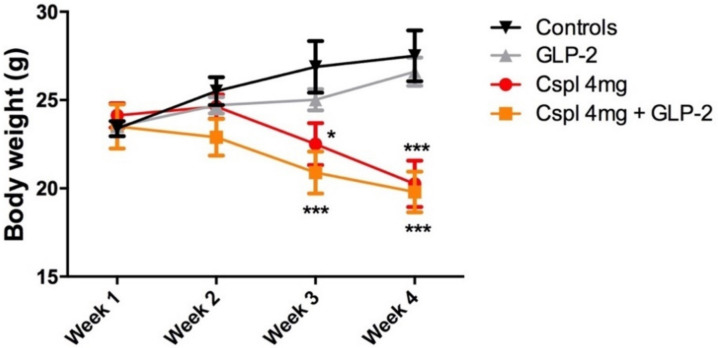
Time course of the bodyweight growth during 4 weeks of treatment. The mice were weighed every Thursday before the cisplatin injection. Data are expressed as mean ± SEM. Two-way ANOVA test, post hoc Bonferroni’s test. *** (*p* < 0.001), * (*p* < 0.05) vs. controls. *n* = 4–6 for each group.

Long-term cisplatin administration (4 mg/kg for 4 weeks) caused a significant decrease in body weight as compared to controls (Figure 1). This decrease started 2 weeks after the first injection of cisplatin and was maintained over time throughout the experimental period (*p* < 0.5; *p* < 0.001). Co-administration of [Gly2]GLP-2 did not prevent cisplatin-induced weight loss (*p* < 0.001). [Gly2]GLP-2 administered alone did not alter body weight time course as compared to control mice (Figure 1).

### 2.2. GLP-2 Prevents Cytokines Increase and Partially Ameliorate Mucosal Damage Induced by Cisplatin in the Distal Colon 

Next, we aimed to define whether cisplatin long-term administration induced mucosal lesions in the distal colon and whether these changes could be prevented by GLP-2 co-administration.

**Figure 2:**
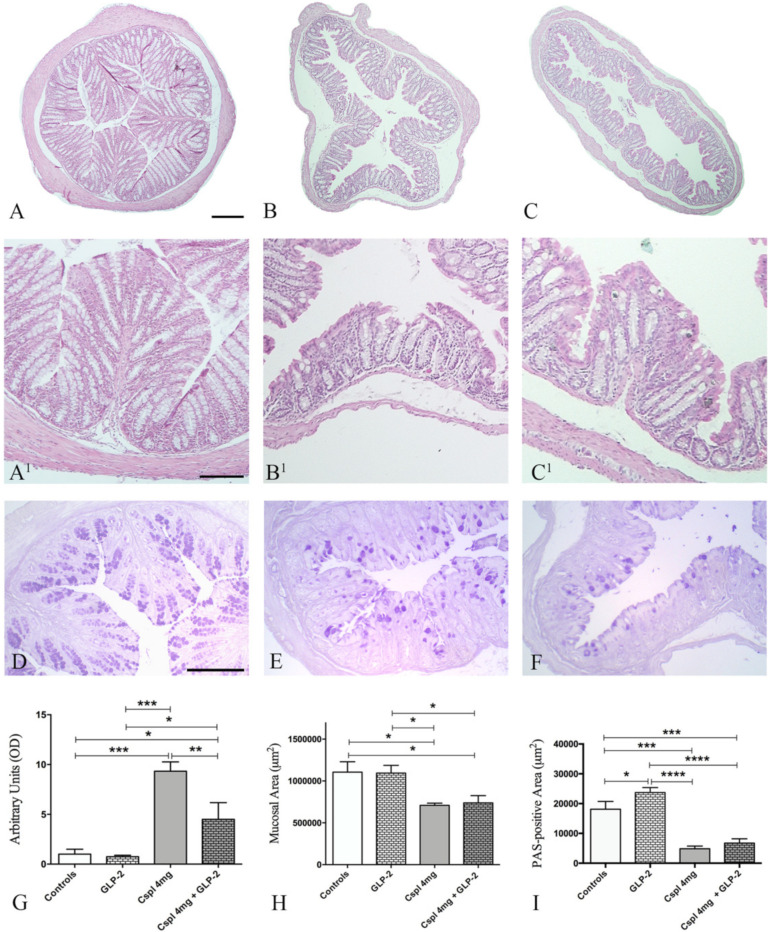
H&E (**A**–**C**,**A**^1^–**C**^1^) and PAS (**D**–**F**) staining of mouse distal colon. Controls (**A**,**A**^1^,**D**), cisplatin (**B**,**B**^1^,**E**) and cisplatin + [Gly2]GLP-2 (**C**,**C**^1^,**F**) group. Scale bar: (**A**–**C**) = 200 μm, (**A**^1^–**C**^1^) = 120 μm; **D**–**F** = 100 μm. Score analysis of mucosal damage (**G**), quantitative analysis of the mucosal area (**H**) and quantitation of PAS staining (**I**). Score analysis (**G**) was performed as described in Materials and Methods. Values are the median ± interquartile range. Non-parametric ANOVA, Kruskal–Wallis, post hoc Dunn’s test. The quantitation of mucosal area (**H**) was done on the whole transverse colonic surface. Data are expressed as mean ± SEM. One-way ANOVA test, post hoc Dunn’s test. The measurement of PAS staining was done on 10 ROIs randomly chosen from the total mucosal area. Data are expressed as mean ± SEM. One-way ANOVA test, post hoc Newman Keuls’ test. **** (*p* < 0.0001), *** (*p* < 0.001), ** (*p* < 0.01), *(*p* < 0.05). *n* = 4–6 for each group.

**Figure 3:**
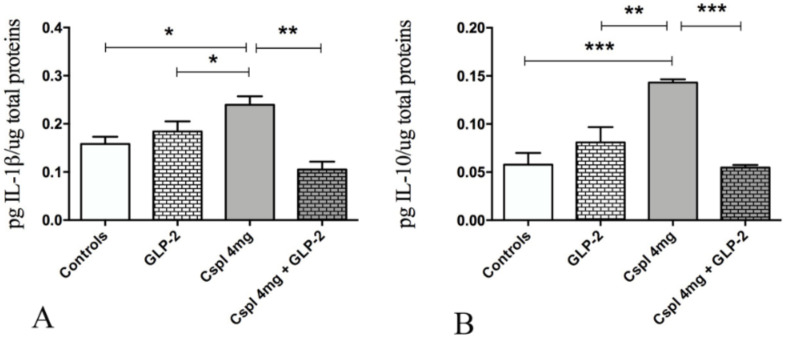
Evaluation of tissue pro- (**A**) and anti- (**B**) inflammatory cytokines. The measurements, performed through ELISA assay were expressed as mean ± SEM of picogram cytokines normalized for the total tissue proteins. One-way ANOVA test, post hoc Newman Keuls’. *** (*p* < 0.001), ** (*p* < 0.01), * (*p* < 0.05). *n* = 4–6 for each group.

Long-term cisplatin administration induced several histological changes in both mucosa and submucosa of the distal colon. In cisplatin-treated mice (Figure 2B,B^1^), the muscularis mucosae showed discontinuity, and mucosal villi appeared unfolded and flattened. Furthermore, the lining epithelium was interrupted, and inter-cryptic distances were reduced and showed an irregular profile as compared to controls. Moreover, in cisplatin-treated mice, the lamina propria exhibited increased inflammatory cell infiltrate as compared to control (Figure 2B,B^1^). Quantification of mucosal damage using a semi-quantitative composite score revealed that cisplatin induced a significant increase in the score as compared to control (Figure 2G) (*p* < 0.001). Moreover, cisplatin induced a 32% significant decrease of the mucosal surface (Figure 2C,C^1^,H) (*p* < 0.05) and a 74% significant reduction of the PAS staining area (Figure 2I) (*p* < 0.001). Cisplatin significantly increased tissue expression of IL-1β and IL-10 cytokines. GLP-2 co-administration partially counteracted the cisplatin-induced increase in the histological damage score (Figure 2G). This effect was associated with a significant reduction in cisplatin-induced increase in IL-1β and IL-10 (Figure 3) (*p* < 0.001; *p* < 0.01). However, GLP-2 co-administration did not significantly reduce mucosal lesions defined as mucosal area and PAS staining (Figure 2H,I).

Finally, we investigated whether cisplatin-induced inflammation and GLP-2 administration modulated the VIP expression.

**Figure 4:**
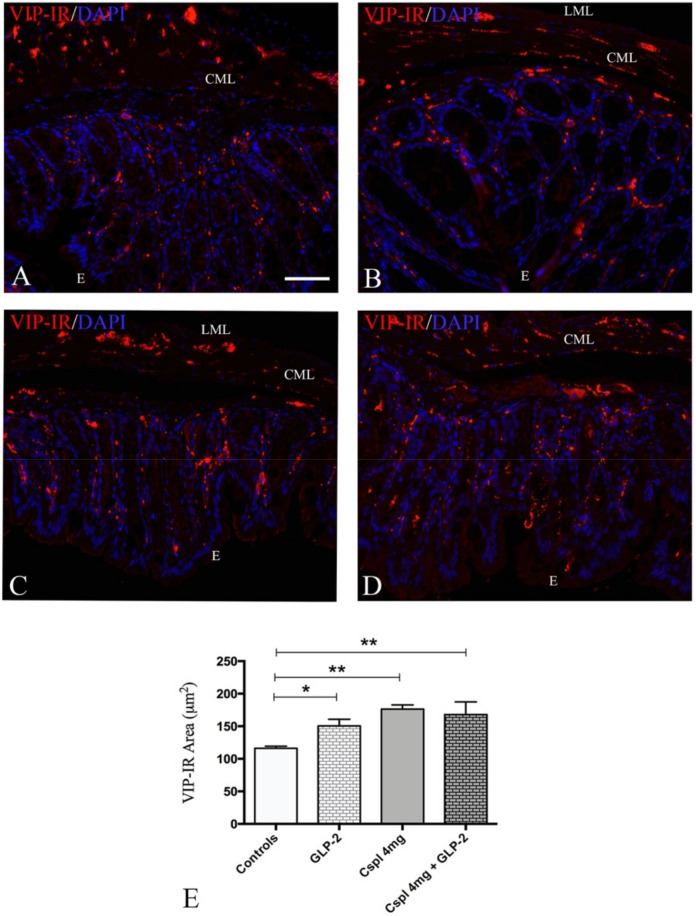
VIP labeling distribution (**A**–**D**) and quantitation (**E**) in colonic mucosa. Controls (**A**), [Gly2]GLP-2 (**B**), cisplatin (**C**), and cisplatin + [Gly2]GLP-2 (**D**) group. The VIP labeling appeared as small granules located in the fiber varicosities along the villi’s axis and under the epithelium. DAPI labeling stained the nuclei. Scale bar = 50 μm. (**E**). 40 regions of interest (ROIs) were randomly chosen to analyze at least 24,000 μm^2^ of tissue. Results are expressed as mean ± SEM. One-way ANOVA test, post hoc Newman Keuls’. ** (*p* < 0.01), * (*p* < 0.05). *n* = 4–6 for each group. E = Epithelium; CML = circular muscle layer; LML = longitudinal muscle layer.

VIP-IR structures were detected in the mucosa as small granules along the villi and lining the epithelium, likely corresponding to the nerve varicosities (Figure 4). The VIP-IR quantitation (Figure 4E) revealed a significant increase in VIP expression in cisplatin and cisplatin + GLP-2 co-treated groups compared to controls (Figure 4E) (*p* < 0.01). Moreover, a significant increase in VIP-IR structures was observed in GLP-2 group in comparison to controls (Figure 4E) (*p* < 0.05). 

### 2.3. GLP-2 Significantly Prevents Neuron and Glia Loss in the Myenteric Plexus Induced by Cisplatin in the Distal Colon 

In the second part of the study, we aimed at characterizing whether long-term cisplatin treatment induced changes in the myenteric plexus and whether GLP-2 co-administration could prevent these changes.

**Figure 5:**
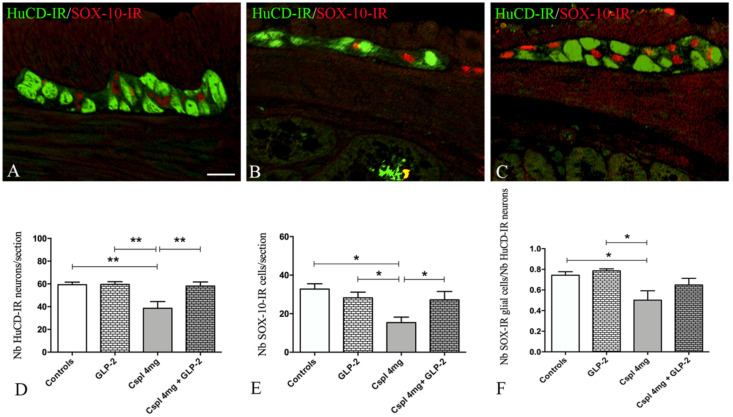
HuCD (green) and SOX-10 (red) double-labeling in the myenteric ganglia (**A**–**C**). Controls (**A**), cisplatin (**B**) and cisplatin + [Gly2]GLP-2 (**C**) group. HuCD-IR neurons appeared larger and more numerous in controls and in co-treated mice (**A**,**C**) compared to those in cisplatin-treated mice (**B**). SOX-10-IR enteric glial cells (EGCs) are in close apposition to the labeled neurons in control and co-treated mice (**A**,**C**) but dispersed and less numerous in cisplatin-treated mice (**B**). Scale bar = 50 μm. Quantitation of the total neuron and EGCs number (**D**,**E**); EGCs/neurons ratio (**F**). Measurements were performed considering the well-oriented ganglia. Data are expressed as mean ± SEM. One-way ANOVA test, Post hoc Newman Keuls’. ** (*p* < 0.01), * (*p* < 0.05). *n* = 4–6 for each group.

**Figure 6:**
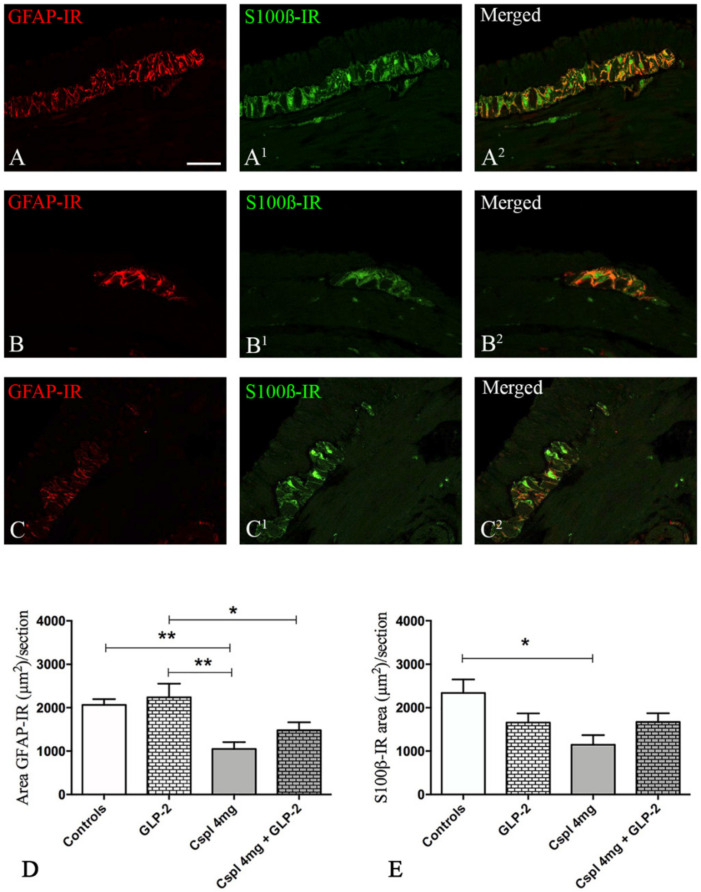
GFAP/ S100β double labeling in the myenteric ganglia. Controls (**A**–**A**^2^), cisplatin (**B**–**B**^2^) and cisplatin + [Gly2]GLP-2 (**C**–**C**^2^) group. GFAP-IR was distributed in the cytoplasm, while S100β -IR was detected either in the cytoplasm or in the nucleus. Quantitation of GFAP- and S100β-IR in the myenteric ganglia. The GFAP (**D**) and S100β (**E**) labeling were measured in well-oriented ganglia, following the criteria described in the Materials and Methods section. Scale bar = 30 μm. Data are expressed as mean ± SEM. One-way ANOVA test, post hoc Newman Keuls’. ** (*p* < 0.01), * (*p* < 0.05). *n* = 4–6 for each group.

First, we characterized the impact of cisplatin upon the number of myenteric neurons and EGCs in the distal colon using HuCD antibody, a pan-neuronal marker (green) and SOX-10 antibody (red) a marker of EGCs (Figure 5A–C). In cisplatin-treated mice, we observed a significant decrease in HuCD-IR cells per section as compared to controls (Figure 5D) (*p* < 0.01) that was completely prevented by GLP-2 co-administration (Figure 5D). In addition, cisplatin-treated mice also exhibited a significant reduction in the number of SOX-10-IR cells as compared to control (Figure 5E) (*p* < 0.05). GLP-2 co-administration also prevented glial cell loss induced by cisplatin (Figure 5E). Finally, the EGCs/neurons ratio (Figure 5F) was significantly decreased in the cisplatin group as compared to control (*p* < 0.05), and this reduction was also prevented by GLP-2. We next aimed at determining whether changes induced by cisplatin and GLP-2 in EGCs were also associated with changes in the expression of key glial markers, i.e., GFAP and S100β. The expression of the GFAP protein was mainly detected in the processes of EGCs (in red), while the S100β protein was in the cytoplasm (in green) of the EGCs (Figure 6A–C^2^). In cisplatin-treated mice, we observed a significant decrease in GFAP- and S100β-IR areas as compared to controls (*p* < 0.05) (*p* < 0.01) (Figure 6D). Co-administration of the GLP-2 did not prevent these decreases.

We further investigated whether changes in neuronal population induced by cisplatin and the effects of GLP-2 upon enteric neurons were associated with specific neuronal subpopulations, i.e., the cholinergic, nitrergic and vipergic. 

**Figure 7:**
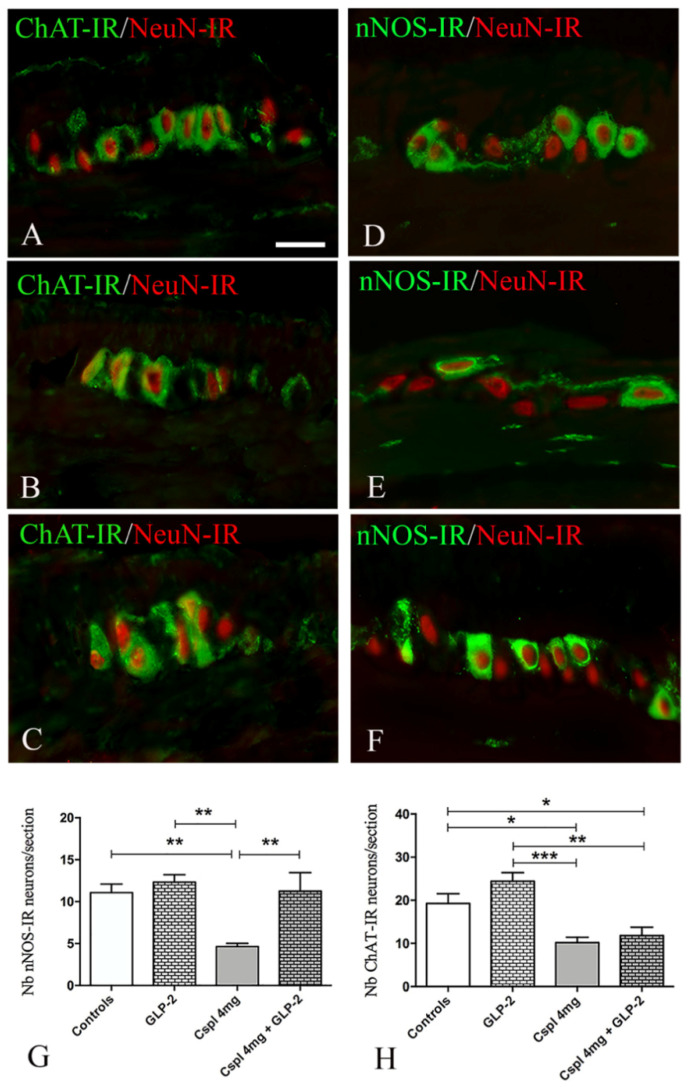
ChAT/NeuN (**A**–**C**) and nNOS/NeuN (**D**–**F**) double labeling in myenteric ganglia. Controls (**A**,**D**), cisplatin (**B**,**E**) and cisplatin + [Gly2]GLP-2 (**C**,**F**) groups. ChAT and nNOS labeling were detected mainly in the neuronal body and in few nerve fibers; NeuN-IR was always observed in the nucleus. Scale bar = 25 μm. Quantitation of the nitrergic and cholinergic neurons number (**G**,**H**). Data are expressed as mean ± SEM. One-way ANOVA test, post hoc Newman Keuls’. *** (*p* < 0.001), ** (*p* < 0.01), * (*p* < 0.05). *n* = 4–6 for each group.

NeuN-IR (red) was used here to identify the neuronal bodies. ChAT labeling (Figure 7A–C, in green) appeared as small granules located in the cytoplasm and processes of several neurons. nNOS labeling (Figure 7D–F, in green) was evenly distributed in the cytoplasm of some neurons. In cisplatin-treated mice, a significant decrease in the number of nNOS-IR neurons per section was observed as compared to controls (Figure 7G) (*p* < 0.01). Co-administration of GLP-2 prevented cisplatin-induced effects upon nNOS neurons (Figure 7G). In cisplatin-treated mice, a significant decrease in ChAT-IR neurons was also observed as compared to controls (Figure 7H) (*p* < 0.05). However, GLP-2 co-administration did not prevent the ChAT-IR neuronal loss induced by cisplatin (Figure 7H). 

**Figure 8:**
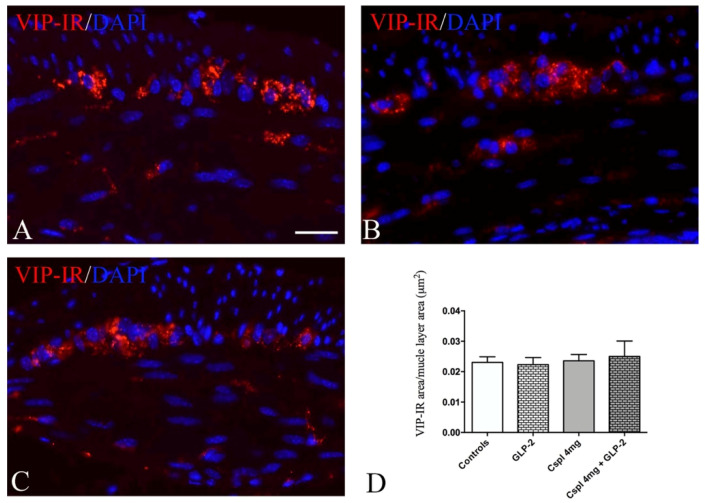
VIP labeling in muscle wall (**A**–**C**). Controls (**A**), cisplatin (**B**) and cisplatin + [Gly2]GLP-2 (**C**) group. VIP labeling was detected as small granules located within the ganglia and muscle layers. DAPI labeling stained the nucleus. Scale bar = 20 μm. Quantitation of VIP-IR structures (**D**). The assessment of VIP expression was performed considering the whole cross-section. Data are expressed as mean ± SEM. One-way ANOVA test, post hoc Newman Keuls’. No significance. *n* = 4–6 for each group.

VIP labeling (Figure 8A–C) appeared as small granules distributed in the myenteric plexus and muscle layers. Quantitation of the VIP-IR structures showed no difference among all the groups of mice (Figure 8D) in myenteric plexus and muscle layers. 

## 3. Discussion

The present results demonstrated that cisplatin greatly affects the distal colonic wall integrity, inducing a significant reduction in mucosa thickness and mucous production and causing inflammation. Furthermore, cisplatin chronic treatment considerably alters the ENS, inducing the loss of both enteric neurons and EGCs. Notably, GLP-2 prevents neuronal and glial losses in a phenotype-dependent manner. Indeed, the hormone specifically protects the nitrergic neuronal subpopulation compared to the cholinergic one and prevents the loss of SOX-10-IR EGCs without significantly reverting the decrease of GFAP and S100β expression. GLP-2 also normalizes the levels of pro- and anti-inflammatory cytokines but does not counteract the mucosal damage. Interestingly, both cisplatin and the GLP-2 significantly increased the VIP expression in the mucosa.

The cisplatin chronic treatment causes a significant loss of body weight that was unaffected by the co-administration of the GLP-2. The weight loss is likely due to the GI mucosal damage [8,12,13] responsible for a defective food intake (anorexia) and nutrient absorption [34]. It is well known that platinum-based treatments induce heavy gastroenteric mucositis [7,35]. Although GLP-2 has been shown to exert effective protection in experimental enteric mucositis induced by different treatments [27,36], in our experimental conditions, the hormone did not protect the mucosa. Nonetheless, it has to be pointed out that none of the previously mentioned studies tested the GLP-2 during long-term exposure to chemotherapy drugs, and yet different outcomes have been reported depending on which GLP-2 form (analogs vs. native), dosage and administration timing were used [27,32,35,36,37,38]. Thus, we suppose that the four weeks exposition to cisplatin results in a mucosal injury too intense for being counteracted by the GLP-2 co-administration. Another important aspect to be considered is the possible modulation of the GLP-2R expression following cisplatin treatment. It has been reported that inflammation causes a decrease in the GLP-2R mRNA expression in mouse and human colon [39]. Since we observed an intense mucosal inflammatory infiltrate in cisplatin-treated group insensitive to GLP-2 co-treatment, we speculate that in our conditions, the GLP-2R expression is also reduced and, consequently GLP-2-beneficial antiapoptotic and proliferative actions are impaired. 

Cisplatin caused a significant increase of IL-1β and IL-10 levels that was prevented by GLP-2 co-administration. Further, and in agreement with the literature [40], we observed that GLP-2 significantly increased VIP expression in the mucosa. The question was whether these two events are correlated. Literature data report that an increase in the number of VIP-IR submucosal neurons is associated with a decrease in inflammatory cytokine levels in murine models of colitis treated with GLP-2 [29,30,39,40]. Thus, we hypothesize that, in our experimental conditions, GLP-2’s effect on cytokines levels is mediated by the VIP increase. On the other hand, VIP expression was also enhanced in cisplatin-only-treated mice, which showed a consistent inflammation. In agreement with the report of Ekblad and Bauer [41], we assume that inflammation per se is able to increase the peptide production. 

An important finding of our study is that repeated cisplatin injections affected both myenteric neurons and EGCs and that the estimation of the glia cells/neurons ratio showed greater impairment of the former with respect to neurons. The neurotoxic effects induced by platinum-based treatments are well known. In the last years, a growing number of experimental and clinical data highlight the ENS as a main target of chemotherapy-induced toxicity. ENS alterations have been associated with GI dysmotility in animal models [3,5,8,9,10,11,12,13,14,42] and humans [3,43] and correlated with the long-lasting and distressing GI side effects experienced by several patients undergoing chemotherapy [4,44]. 

We presently show that cisplatin induces an imbalanced neuronal loss characterized by a decrease of the nNOS and ChAT myenteric neurons without changes in VIP-IR structures. 

The expression of ChAT, nNOS and VIP has been investigated in different experimental models using diverse chemotherapy treatments [9,11,13,45,46]. For instance, the repeated administration of 5-fluorouracil caused a global loss of myenteric neurons associated with a selective decrease of ChAT- and nNOS-IR neurons and a delayed and long-lasting colonic contractility [46]. The administration of irinotecan resulted in a global loss of myenteric neurons associated with an increased proportion of ChAT-IR neurons and with diarrhea [45]. Chronic treatments with cisplatin or oxaliplatin caused nNOS-IR neuronal loss and delay of colonic motility and upper intestinal transit [11,12,13]. Finally, in line with our observation, Vera et al. reported no alteration of the VIP-IR in the colonic muscle wall during chronic cisplatin treatment in rats [9]. Regardless, it would be interesting to gain insight into the reason why these neuronal subpopulations show different sensitivity to the anticancer agents. In this regard, it has been postulated that membrane transporters are involved in platinum-based neurotoxicity [47]. Thus, it might be that each of these drugs enters or exits the enteric neurons through specific membrane transporters (i.e., the copper efflux transporter), which might be differentially distributed among the neuronal subpopulations [47]. 

Concerning the GLP-2, our findings show that the hormone prevents the cisplatin-induced loss of myenteric neurons, specifically protecting the nitrergic subpopulation compared to the cholinergic one. Few studies have dealt with GLP-2 neuroprotection. Ekblad et al. showed that the addition of GLP-2 in ENS primary cultures prevented the neuronal loss caused by mast cell degranulation [33]. We previously demonstrated that the administration of GLP-2 in mice chronically treated with cisplatin prevented the loss of nitrergic neurons in the gastric fundus [12]. Present data are in line with this finding and confirm that GLP-2 plays a specific role in neuronal protection upon the nitrergic component, regardless of the GI region involved. A functional relationship between GLP-2 and nNOS has recently been reported, as the addition of GLP-2 to in vitro gastric strips increased nNOS expression and modulated nitrergic neurotransmission [48]. The GLP-2 nitrergic neuroprotection is possibly due to NO production. Concerning this, Sandgren et al. [49] showed that NO addition to myenteric neurons’ primary culture enhanced cell survival. However, the action of NO within the ENS is still controversial. Basically, it is known that NO enhances neuronal survival in some circumstances, while it promotes neuronal death in others [50]. Nevertheless, the exact mechanisms underlying neuroprotective action of GLP-2 cannot be determined based on the present experiments, but it represents an interesting research field worth developing. 

Compared to the platinum-related enteric neurotoxicity, glial alterations have not been thoroughly studied along the intestine, and only Nurgali and co-workers showed that oxaliplatin chronic treatment increased the levels of S100β and decreased those of GFAP within the myenteric plexus of the mouse ileum and distal colon [11,14]. Conversely, in the present study, we demonstrated that cisplatin administration induced a reduction of both GFAP and S100β expression. In addition, for the first time, we showed that the cisplatin chronic administration caused a loss of the SOX-10-positive EGCs. As this marker has been found co-expressed with the majority of GFAP- and S100β-IR myenteric EGCs (74% and up to 95%, respectively [51]), we consider Sox-10 reliable for labeling almost all EGCs. The differences between our findings and those of Nurgali’s group could be due to a diverse effect exerted by oxaliplatin and the timing of treatment used in their animal model. Since we hypothesized that chronic cisplatin treatment caused a general loss of ECGs, as identified with SOX-10, a decrease in GFAP- and S100β is the logical consequence. Interestingly, GLP-2 was able to protect the SOX-10-IR cells in co-treated mice, but surprisingly, this effect was not accompanied by a recovery of GFAP and S100β expression. A possible explanation of these results is that GLP-2 prevents EGCs death, but it is not able to preserve their functional and structural integrity [33].

It is well known that the EGCs exert neuroprotective actions [52,53,54], and their involvement in intestinal pathologies has been reported by several authors [51,53,55]. Thus, in our model, the loss of EGCs might expose the neurons to aggressive agents and, consequently, cause neuronal death. Alternatively, it can be speculated that cisplatin might induce an unspecific toxicity upon the ENS, thus involving both neurons and EGCs. The literature lacks information concerning the mechanism by which cisplatin and other platinum-based drugs induce damage to the ENS. McQuade and co-workers [42] have proposed oxidative stress as a key player in enteric neuropathies, while no data are available to explain the cellular mechanism of chemotherapy-induced gliopathy. It is also possible that oxidative stress involves EGCs. 

In summary, this study demonstrates that the GLP-2 analog counteracts most of the cytotoxic effects induced by chronic cisplatin administration in the mouse distal colon. Although the [Gly2]GLP-2 partially prevents the mucosal injuries, it intervenes in the local inflammation, avoiding the increase of pro- and anti-inflammatory cytokines levels, likely through the enhancement of VIP expression. Furthermore, the GLP-2 analog prevents both myenteric neurons and EGCs loss caused by cisplatin, exerting selective neuroprotection on the nitrergic component.

In conclusion, this work provides evidence that the administration of the GLP-2 analog during the cisplatin regimen might be an effective strategy to overcome the drug-induced colonic toxicity. 

## 4. Materials and Methods

### 4.1. Animals Experimental Design

C57BL/6 female mice (18–22 g; *n* = 24) were obtained from Harlan Laboratories (Correzzana, Italy). They were allowed to acclimatize to the climate- and light-controlled animal facility for one week, and before starting the treatment, the mice were randomly divided into five groups: control group (controls; *n* = 3); vehicle-treated group (vehicle, *n* = 3) (both controls and vehicle mice were housed in the same cage); [Gly2]GLP-2 treated group (GLP-2; *n* = 6); cisplatin 4 mg-treated group (Cspl 4 mg; *n* = 6); and Cisplatin 4 mg + [Gly2]GLP-2-treated group (Cspl 4 mg + GLP-2; *n* = 6). Each of the latter three groups was housed in a separate cage. During the treatment, 2 cisplatin-treated mice died. 

The experimental protocol was designed in compliance with the guidelines of the European Communities Council Directive 2010/63/UE and the recommendations for the care and use of laboratory animals approved by the Animal Care Committee of the University of Florence, Italy; the animals lived under standard conditions, as previously described [12]. The general condition of the mice was assessed daily, and the bodyweight was weekly registered. Specifically, the bodyweight was measured at the animal arrival and every Thursday before the cisplatin injection. The mice were daily treated with [Gly2]GLP-2 (Caslo Laboratory, Lyngby, Denmark), a degradation-resistant GLP-2 analog with a longer half-life in vivo than the native peptide, dissolved in sterile saline solution and injected i.p. at the concentration of 50 μg kg^−1^. The [Gly2]GLP-2 dosage used corresponds to that recommended for the treatment of Short Bowel Syndrome [31,56]. Cisplatin injectable solution (kindly provided by Dr. T. Falai, SOD of Pharmacy, AOU Careggi, Florence, Italy) was intraperitoneally (i.p.) administered twice a week (on Monday and Thursday) at a dosage of 4 mg kg^−1^. To prevent cisplatin-induced nephrotoxicity, 1 mL of saline was injected subcutaneously just before each cisplatin injection, and the mice, aside from untreated controls, received the same number of injections. The [Gly2]GLP-2 treatment was commenced on Monday of the first week of treatment simultaneously with the first weekly cisplatin administration. The treatments were performed for 4 weeks. On Monday of the fifth week, 3 days after the last cisplatin injection, the animals were sacrificed. Samples from the distal colon were taken from the animals for all analyses.

### 4.2. Tissue Sampling 

The abdomen was immediately opened, and the distal colon was quickly removed. The content of the excised segments was washed with ice-cold physiological saline solution, and segments of about 0.5 cm in length were cut. Some colon specimens were frozen at −80 °C for future molecular biology experiments; others were fixed in 4% paraformaldehyde in 0.1 M phosphate-buffered saline (PBS) pH 7.4 and then embedded in paraffin for morphological analysis. 

### 4.3. Morphological Studies 

#### 4.3.1. Histology and Histochemistry 

Full-thickness cross-sections (5 μm thick) stained with H&E or with PAS reaction were used for semi-quantitative scoring system and quantitative morphometric analysis. All sections were stained in a single session to minimize artefactual staining differences, and two sections per animal were analyzed. Score analysis adapted from Vera et al., 2011 [9] was performed by two blinded observers (P.N., M.G.V.) using the following four criteria: loss of crypt architecture (graded 0–3, normal, mild, moderate, severe); extent of inflammatory cells infiltrate in lamina propria (graded 0–3, normal, mild, moderate, severe); loss of epithelial integrity (graded 0–1, presence–absence); loss of muscularis mucosa continuity (graded 0–1, presence–absence). Thus, a numerical score of 0–8 was assigned to each animal. The total mucosal area and mucus secretion were analyzed using ImageJ software (NIH, Bethesda, ML, USA) on digitalized images acquired at 10× and 40× objectives, respectively, with a microscope fitted with a camera (Leica DFC310 F× 1.4-megapixel camera, Leica Microsystems, Mannheim, Germany). Mucin content was quantified on 10 photomicrographs or regions of interest (ROIs) randomly taken for each section measuring the PAS-positive areas, exclusively selected using the photograph’s threshold values, while for mucosal area, the measurement was carried out on the whole mucosal surface.

#### 4.3.2. Immunohistochemistry

The paraffin-embedded sections, once deparaffinized and rehydrated as usual, were treated for antigen retrieval in some cases for 20 min at 90–92 °C in Tris buffer (10 mM) with EDTA (1 mM, pH 9.0), in others for 90 s at 110 °C with antigen retrieval solution (Dako, Santa Clara, CA, USA) followed by cooling to room temperature (RT). The sections were then washed in PBS, blocked with 1.5%, 5% bovine serum albumin (BSA, Applichem, Darmastad, Germany) in PBS or in blocking solution (Dako) for 20 min/1 h at RT to minimize no-specific binding. The primary antibodies (see Table 1) were diluted in BSA 1.5% PBS (5% BSA PBS was used only for ChAT labeling) and were incubated overnight at 4 °C. The omission of the primary antibodies was used as negative control. The next day, the sections were incubated for 2 h at RT in the dark with appropriate fluorochrome-conjugated secondary antibodies diluted in BSA 1.5% PBS or 5% BSA PBS for ChAT labeling. Subsequently, the specimens were rinsed three times with PBS and then mounted with an aqueous medium (Mountant Permafluor, Thermo Scientific, Rockford, IL, USA). The double labeling was performed in some sections as follows: nNOS/NeuN, ChAT/NeuN, HuCD/SOX-10 and GFAP/S100β. The HuCD/SOX-10 and GFAP/S100β-labeling fluorophores were visualized using a Nikon (Tokyo, Japan) A1R confocal microscope, and appropriate laser wavelength and filters, with 60/1.4 objective and full-size images were recorded with NIS (Nikon) software. The image acquisition was performed in collaboration with the Confocal Cellular and Tissutal Imaging Core Facility of Nantes University (MicroPICell). The nNOS/NeuN, ChAT/NeuN labeling was observed under an epifluorescence Zeiss Axioskop microscope (Zeiss, Oberkochen, Germany) using excitation filters for Alexa 594 red and Alexa 488 green. The fluorescence images were captured using a Leica DFC310 FX 1.4-megapixel digital camera equipped with the Leica software application suite LAS V3.8 (Leica Microsystems). The total number of HuCD-and SOX-10-IR cells, and the number of the nNOS- and ChAT-IR neurons was evaluated within the well-oriented myenteric ganglia along the entire section by two observers (PN, MGV) blindly to each other, and the results are expressed as the number of neuronal and glial cell bodies per sections ± S.E.M (two sections per animal; 4–6 animals/group). The quantitation of GFAP-, S100β- and the VIP-IR structures (nerve fibers and cell bodies) was morphometrically assessed on digitized images acquired with a 60× and 20× objective by using threshold tool of ImageJ software (2 section/animal; 4–6 animals/group).

### 4.4. Biochemical Study 

#### 4.4.1. Cytokine Enzyme-Linked Immune-Absorbent Assay

Distal colon samples previously stored at −80 °C were thawed slowly at 0 °C and then homogenized with a tissue homogenizer (Ing. Terzano, Milan, Italy) in a cold lysis buffer: 10 mmol/L Tris/HCl, pH 7.4, 10 mmol/L NaCl, 1.5 mmol/L MgCl_2_, 1% Triton X-100, 0.1% SDS, added with 106 Sigmafast Protease Inhibitor cocktail tablets (Sigma, St Louis, MO, USA). The homogenized tissue was centrifuged at 14,000× *g* for 20 min at 4 °C to obtain the supernatants, which were subsequently collected. The total protein content was measured spectrophotometrically using micro-BCA Protein Assay Kit (Thermo scientific, Rockford, IL, USA) for calibration. Proteins concentration of IL-1β and IL-10, such as the main pro- and anti-inflammatory cytokines, was determined in the tissue supernatants using enzyme-linked immunoabsorbent assay (ELISA) kits (BioLegend, San Diego, CA, USA) according to the manufacturer’s instructions.

#### 4.4.2. Data Analysis and Statistical Test

The results obtained from control and vehicle groups were pooled together and referred to as controls. Statistical analyses were performed using GraphPad Prism software (GraphPad, San Diego, CA, USA). Statistical differences between mean values were analyzed by analysis of variance (one-way or two-way ANOVA) followed by Newman–Keuls to compare more than two groups. Statistical significance was defined as a *p*-value less than 0.05. When the data were not representative of a normal distribution, non-parametric test Kruskal–Wallis one-way ANOVA followed by Dunn’s test was used.

## Figures and Tables

**Table 1:** Primary and secondary antisera used in immunohistochemistry.

Antigen	Species	Source	Concentration
**Primary Antisera**			
nNOS	Rabbit	Millipore (Bedford, MA, USA)	1:2000
NeuN	Mouse	Millipore	1:200
ChAT	Goat	Millipore	1:200
VIP	Mouse	Santa Cruz Biotech (Santa Cruz, CA, USA)	1:200
SOX-10	Goat	SantaCruz	1:500
HuCD	Rabbit	SantaCruz	1:200
S100β	Rabbit	Dako (Dako, Santa Clara, CA, USA)	Ready to use
GFAP	Mouse	Sigma (St Louis, MO, USA)	1:300
**Secondary Antisera**			
Alexa Fluor 488	Goat	Invitrogen (Carlsbad, CA, USA)	1:333
Alexa Fluor 594	Mouse	Jackson ImmunoResearch (Ely, Cambridgeshire, UK)	1:333
Alexa Fluor 488	Rabbit	Jackson ImmunoResearch	1:333
Anti-mouse C3	Donkey	Jackson ImmunoResearch	1:500
Anti-rabbit-FP488	Donkey	Interchim (Montluçon, France)	1:200
